# The potential role of phytochemicals in wholegrain cereals for the prevention of type-2 diabetes

**DOI:** 10.1186/1475-2891-12-62

**Published:** 2013-05-16

**Authors:** Damien P Belobrajdic, Anthony R Bird

**Affiliations:** 1 Commonwealth Scientific & Industrial Research Organisation (CSIRO) Food Futures National Flagship, GPO BOX 10041, Adelaide, SA, 5000, Australia; 2 CSIRO Animal Food & Health Sciences, Adelaide, SA, 5000, Australia

**Keywords:** Wholegrain, Phytochemical, Type-2 diabetes, Oxidative stress, Inflammation

## Abstract

Diets high in wholegrains are associated with a 20-30% reduction in risk of developing type-2 diabetes (T2D), which is attributed to a variety of wholegrain components, notably dietary fibre, vitamins, minerals and phytochemicals. Most phytochemicals function as antioxidants in vitro and have the potential to mitigate oxidative stress and inflammation which are implicated in the pathogenesis of T2D. In this review we compare the content and bioavailability of phytochemicals in wheat, barley, rice, rye and oat varieties and critically evaluate the evidence for wholegrain cereals and cereal fractions increasing plasma phytochemical concentrations and reducing oxidative stress and inflammation in humans. Phytochemical content varies considerably within and among the major cereal varieties. Differences in genetics and agro-climatic conditions explain much of the variation. For a number of the major phytochemicals, such as phenolics and flavanoids, their content in grains may be high but because these compounds are tightly bound to the cell wall matrix, their bioavailability is often limited. Clinical trials show that postprandial plasma phenolic concentrations are increased after consumption of wholegrain wheat or wheat bran however the magnitude of the response is usually modest and transient. Whether this is sufficient to bolster antioxidant defences and translates into improved health outcomes is still uncertain. Increased phytochemical bioavailability may be achieved through bio-processing of grains but the improvements so far are small and have not yet led to changes in clinical or physiological markers associated with reduced risk of T2D. Furthermore, the effect of wholegrain cereals and cereal fractions on biomarkers of oxidative stress or strengthening antioxidant defence in healthy individuals is generally small or nonexistent, whereas biomarkers of systemic inflammation tend to be reduced in people consuming high intakes of wholegrains. Future dietary intervention studies seeking to establish a direct role of phytochemicals in mediating the metabolic health benefits of wholegrains, and their potential for mitigating disease progression, should consider using varieties that deliver the highest possible levels of bioavailable phytochemicals in the context of whole foods and diets. Both postprandial and prolonged responses in systemic phytochemical concentrations and markers of inflammation and oxidative stress should be assessed along with changes related to health outcomes in healthy individuals as well as those with metabolic disease.

## Metabolic disease and protective role for wholegrains

Type-2 diabetes (T2D) is a major health problem worldwide. Rates are increasing alarmingly in many countries and the global incidence is predicted to rise from 366 million people to about 552 million in the next two decades [1,2]. It is a leading cause of death and disability globally and carries a considerable socioeconomic burden, especially in low and middle income settings [2-5]. Cost-effective mitigation strategies rather than containment are therefore of paramount importance. The initiation and progression of T2D and related chronic metabolic disorders is governed by a complex interplay of genetic and multiple lifestyle influences of which diet is a major and modifiable high exposure risk factor. Dietary change has proven successful in both preventing and managing diabetes and, when combined with other lifestyle modifications, such as regular exercise and weight loss, is more effective than pharmacological interventions [6].

Dietary patterns featuring wholegrain cereals are associated with reduced risk of T2D [7-10]. Systematic reviews and meta-analyses of large, prospective studies consistently demonstrate that frequent consumption of wholegrain foods improves metabolic homeostasis and delays or prevents the development of T2D and its complications in a variety of cohorts, albeit mostly of European ancestry [11-18]. Two to three serves daily of wholegrain foods reduced the risk of T2D by 20-30% compared to about 1 serve a week [12,13,15,18]. Randomised, controlled dietary studies in humans and other experimental research provides evidence of a causal relationship between wholegrain consumption and diabetes prevention [15,18]. Furthermore, wholegrain foods improve indices of diabetes risk, including glycemic control, fasting plasma insulin and glucose, and insulin sensitivity and also aid in the management of those individuals with or at high risk of developing T2D [13,16,19-21].

### Mechanisms by which wholegrains might protect against type-2 diabetes

Understanding the mechanisms by which wholegrains prevent or delay the onset and progression of T2D is pivotal to developing effective diabetes prevention options. The components of wholegrains which are responsible for protecting against diabetes have not been clearly identified but the high nutrient and fibre contents in general, as well as the physical structure of wholegrains are considered leading contenders [15,22,23]. Prospective studies show that T2D risk is inversely related to cereal fibre intake [18] and that cereal fibre accounts for much of the reduction in diabetes risk associated with wholegrain intake [24]. Dietary fibre is concentrated primarily in the bran layer of grains and it is this fraction which is more strongly associated with reduction in risk of T2D [17]. Most but not all wholegrains are high in fibre [25] and individual wholegrains differ markedly in the types and hence physiological properties of fibres they contain. Viscous soluble fibres, such as those in oats and barley, slow available carbohydrate assimilation and dampen postprandial glycemic and insulinemic responses [26]. However most observational studies provide evidence of a protective role for insoluble rather than soluble fibres [27]. The likely explanation is that insoluble fibre is simply serving as a marker of an intact (grain) food structure. Foods and diets rich in carbohydrates that are rapidly digested and absorbed have adverse consequences for metabolic health [28-33]. Refinement of cereal grains removes the protective bran layer and greatly increases starch availability. However, not all wholegrain foods elicit a moderate glycemic response [34]. Although wholegrain foods may contain intact, cracked, broken or flaked kernels, most commercially processed cereal foods consist of ground and reconstituted wholegrain products [25].

Wholegrains contain a plethora of minerals, vitamins and phytochemicals [35] and it is often difficult to ascribe protective effects on metabolic health to any one particular constituent, such as fibre. One of the primary pathogenic factors leading to insulin resistance, β-cell dysfunction, impaired glucose tolerance and ultimately T2D is oxidative stress [36-38]. This mechanism has been implicated as the underlying cause of both the macrovascular and microvascular complications associated with T2D [39]. Furthermore, the cells and tissues of people with metabolic syndrome and T2D have an impaired ability to cope with the burden of increased oxidative stress [40-42]. Therefore, dietary components including phytochemicals (non-nutritive, plant bio actives that reduce risk of chronic diseases [25]), and a limited number of micronutrients that function as antioxidants, may prevent the development and progression of metabolic syndrome and T2D by reducing oxidative stress [35]. Furthermore, systemic, low grade inflammation, especially in adipose tissue, is a hallmark of many chronic diseases, including T2D [43]. In addition to their antioxidant properties, some cereal phytochemicals have potent anti-inflammatory actions [44,45] and may thereby modulate diabetes risk by this mechanism as well [43,46,47].

### Phytochemicals in whole grains

Wholegrains generally contain diverse combinations of phytochemicals depending on the type of cereal, location within the grain and how the grain has been processed. The outer structures of grains, in particular the pericarp seed coat and aleurone layers, contain much higher levels of phytochemicals such as phenolic compounds, phytosterols, tocols, betaine and folate, than the germ and endosperm [48]. Phenolic compounds are the most diverse and complex class of phytochemicals in cereal grains [35,49]. They include numerous derivatives of benzoic and cinnamic acids as well as flavonoids, flavones and flavanols, anthocyanidins, avenanthramides, lignans and alkylresorcinols. In most grains phenolic acids are concentrated in the bran and embryo cell walls and exist mostly in an insoluble bound form, free and soluble-conjugated forms being minor entities [25,48]. The phenolic acid content of wholegrains is considered a major contributor to total antioxidant capacity [25]. Other major phytochemicals that occur in wholegrains which may have a role in protecting against diabetes include various carotenoids, notably α- and β-carotene, lutein, β-cryptoxanthin and zeaxanthin, all of which are located mainly in the bran and germ fractions [49]. Aside from some having pro-vitamin A activity, they all function as antioxidants. Other phytochemicals with strong antioxidant capacities include phytate (which chelates prooxidant minerals) and various terpenes and terpenoids (phytosterols and tocols).

To render them palatable, grains are processed by various means including milling, grinding and flaking. Although these treatments may reduce content of phytochemicals, their bioavailability is often increased [45,50,51]. Thermal and bioprocessing too can improve phytochemical bioavailability, especially the latter method, although the results are not always consistent.

Differences among the most economically important cereals in their contents of various micronutrients and phytochemicals are shown in Table 1. Note that the phytochemical values refer to uncooked wholegrains. Wholegrains are normally cooked and are rarely consumed in their unprocessed or raw form. Cooking results in considerable reductions in their phytochemical levels. For instance, quick-cooking wild rice had a much lower total phenolic content (2076 mg ferulic acid equivalent (FAE)/kg) than uncooked wild rice varieties (2472 to 4072 mg FAE/kg) [52].

**Table 1:** The type and concentration of phytohcemicals in a range of wholegrain cereals

**Phytochemical**	**Wheat**	**Barley**	**Rice**	**Rye**	**Oat**
Methionine (g/100g) [48,53-56]	0.17 – 0.24	0.03 – 0.08	0.18 – 0.21	0.18	0.18
Cystine (g/100g) [48,53-56]	0.19 – 0.40	0.06 – 0.2	0.11 – 0.16	0.18	0.18
Selenium (mg/100g) [48,57-60]	0.0003 – 3	0.002 – 0.030	0.0002 – 1.37	0.00014	< 0.10 – 3.3
Folate (mg/100g) [60,61]	0.01 – 0.09	0.5 – 0.8	0.016	0.55 – 0.80	0.05 – 0.06
Choline (mg/100g) [48,62]	27 – 195	6.9 – 11	Unknown	Unknown	2.0 – 2.6
Tocopherols + tocotrienols [48,63-67]	2.3 – 8.0	4.7 – 6.8	0.4 – 0.9	0.4 – 0.7	0.05 – 4.8
Carotenoids (total) (mg gallic acid eq./100g) [48,60]	0.04 – 0.63	0.015 – 0.105	0.014 – 0.077	Unknown	0.031
Polyphenols (mg/100g) [60]	70 – 1459	50 – 196	54 – 313	125 – 255	9 – 34
Phenolic acids (total) (μg/g) [61,64,68]	200 – 900	100 – 550	Unknown	200 – 1080	350 – 874
Phenolic acid (free) (ug/g) [61,64,68]	5 – 39	5 – 23	Unknown	10 – 35	50 – 110
Ferulic acid (total, mg/100g) [48,69]	16 – 213	110 – 120	30	3.9 – 5.0	2.1 – 2.4
Flavanoids (total, mg/100g) [48,69]	30 – 43	12 – 18	Unknown	6.7 – 7.5	5.6 – 8.2
Other (mg/100g)					
Alkylresorcinols μg/g [61,70]	200 – 750	0 – 150	Not present	570 – 3220	Not present
Avenanthramides (mg/100g [71,72]	Not present	Not present	Not present	Not present	4.9 – 27.5
Betaine (mg/100g) [48,60,62,73]	22 – 291	40 – 76	0.5 (brown)	Unknown	11.3 – 100
Phytosterols (mg/100g) [48,74]	57 – 98	90 – 115	Unknown	Unknown	Unknown

### Variation in grain phytochemical content

Wheat, barley, rice, rye and oats vary markedly in the types and amounts of phytochemicals they contain.

a. Wheat

The antioxidant properties of wheat have been attributed primarily to the high phenolic content, principally alkylresorcinols and hydroxycinnamic acids (ferulic, sinapic, and ρ-coumaric acids) that are concentrated in the bran fraction [49,75-77]. The flavonoid concentration in the bound fraction of wheat cultivars has been shown to vary from 97 ± 4 μmol catechin equivalents/100 g (Roane) to 139 ± 17 μmol catechin equivalents/100 g (Superior) [78]. However, there is less variation in total flavonoid content (122 ± 10 μmol catechin equivalents/g (Roane) to 149 ± 17 μmol catechin equivalents/100 g (Superior) [78]) whereas tocopherols and tocotrienols levels vary more than 2-fold (28 to 80 μg/g) among 175 different wheat genotypes from all over the world grown at the same site in Europe [79]. Even greater variation (up to 10-fold variation) was seen in α-tocopherol levels measured in several hundred wheat cultivars grown in the United States [80].

b. Barley

The major phytochemicals in barley are phenolics, tocols and folate. Analysis of a selection of 10 barley lines showed a large variation in the concentration of total phenolics (100 to 550 ug/g) but only minimal variation in folate (500 to 800 ug/g) and total tocols (45 to 70 ug/g) [61]. Our group has recently developed a new variety of barley, BARLEYmax® [81] that has a range of substantiated nutritional and health benefits [82,83]. It has a phenolic content (5 mg/g) which is 40% greater than that of standard cultivars such as Golden Promise (2.9 mg/g) and Torrens (3 mg/g). It also contains levels of tocopherol and tocotrienols (125 μg/g) which are nearly 5 times those of other barley grain varieties (McInerney, JK, Morell, MK and Bird, AR unpublished data).

c. Rice

Brown rice generally is a good source of lipid-soluble antioxidants including ferulated phytosterols (i.e. γ-oryzanol), tocopherols and tocotrienols, although the levels of these phytochemicals vary widely among rice varieties [63]. For instance, tocol concentration ranged from 90 to 220 nmol/g in six varieties of rice [63]. Brown rice may also be a good source of phenolic acids as suggested by the levels reported for the botanically related wild rice (*Zizaniae palustris* and *Zizaniae aquatica;* 2472 to 4072 mg of ferulic acid equivalent (FAE)/kg). These values are substantially higher than that of the mixed sample of white rice, basmati rice and wild rice (1460 mg of FAE/kg) [52]. The total phenolics content of these rices was directly related to their in vitro antioxidant capacity, which was 30 times higher for wild rice than the control (white) rice [52].

d. Rye

Rye contains more alkylresorcinols (568 to 3220 μg/g) than the other major cereal varieties (0 to 750 μg/g). The concentration of alkylresorcinol in rye [70,84] is related to the high level of folate in the grain (0.55 to 0.80 mg/100 g) [64]. Select varieties of rye also contain very high levels of total phenolics (up to 1080 μg/g) but the content of free phenolics is quite low (between 10 to 35 μg/g) [64]. Other phytochemicals, including tocols, polyphenols and ferulic acid are found at low levels in rye [64].

e. Oats

The major phytochemicals present in oats include tocopherols and tocotrienols, phenolic acids, sterols, selenium and avenanthramides (a group of N-cinnamoylanthranilate alkaloids, unique to oats) [85,86]. Tocol levels differ greatly (5 to 48 μg/g) [61,65,66] among oat varieties but generally are comparable to those found in rice and rye (4 to 9 μg/g) and also to the higher levels found in wheat and barley (23 to 80 μg/g) [61]. The range in the total phenolic levels of oats are also similar to those in wheat and rye, however oats contains up to 10-fold higher levels of free and conjugated phenolics. Other phytochemicals, including folate, polyphenols, ferulic acid and flavonoids are present at low levels in oats.

### Major regulators of phytochemical content of cereals: genetics and agro-climatic conditions

The phytochemical content of cereal grains is influenced considerably by genetics and a variety of agro-climatic factors. In rice, the growing environment had a greater effect on tocol and/or sterol esters of ferulic acid levels than did genotype [87,88]. In wheat, genetic variation and agro-climatic conditions are both important but the extent of their influence depends on the phytochemical concerned. In an assessment of over 200 lines of wheat, α-tocopherol levels were influenced by not only varietal differences but also crop year and production site [80]. Fertilization practices, soil type and wheat variety had no influence [80]. Additionally, when eight selected winter wheat genotypes were grown under controlled conditions α-tocopherol levels varied by as much as 3-fold, highlighting the significant contribution of genetic variation [89]. However, studies in Europe show that tocopherol and tocotrienol levels in some wheat varieties are more susceptible to seasonal variation than others [90]. This greater susceptibility to seasonal variation and growing location is also evident in some wheat genotypes for free and conjugated phenolic levels [91]. However, bound phenolics which comprise the greatest proportion of total phenolic acids in wheat, are mostly stable across different growing conditions. Thus, the total phenolic acid content of wheat is mostly influenced by genotype, for instance winter varieties contain up to 2-fold more total phenolic acids (1171 μg/g) than the average level of 175 wheat genotypes (658 μg/g) [92].

### Phytochemical bioavailability

Bioavailability refers to the fraction of ingested phytochemical (or other dietary constituent) which reaches the systemic circulation. More commonly it is defined as the fraction which is absorbed in the gastrointestinal tract. Tracer methods, in which atoms or molecules of the phytochemical within the grain are labelled with an intrinsic radioactive or stable marker, provide the only means for accurately determining bioavailability. Given the challenges of labelling cereal phytochemicals intrinsically, this technique has not been used to measure bioavailability of phytochemicals in cereals. Simpler indirect measures are more commonly used, such as the balance method (intake minus fecal output), incremental area under the postprandial serum concentration curve and incremental urinary excretion. Numerous in vitro methods have also been published however they, understandably, have many limitations [93] aside from questionable validity, and so provide at best only a guide to the bioaccessibility of a phytochemical.

a. Absorption from the small intestine

Bioavailability varies markedly among the different types of phytochemicals. Folate and α-tocopherol are readily absorbed from the small intestine and their bioavailability is independent of dietary fibre content (Table 2) [94,95]. The majority of polyphenols however, are tightly bound to cell walls within the grain matrix thereby greatly limiting their bioavailability in the upper gut [96]. Even if polyphenols are released from the grain matrix during digestion it is unlikely that they will be absorbed in the small intestine as they are too hydrophilic to cross the epithelium by passive diffusion [97]. It is possible that there are apical membrane carriers that facilitate polyphenol absorption however the intestinal transport processes remain largely unknown [97]. Oats contain the highest levels of free, or unbound, phenolics (up to 30% of total phenolics) whereas wheat, barley and rye contain only very low levels (as little as 1.6%) [61]. Thus specific varieties of oats have the greatest potential to raise postprandial plasma phenolic concentration and antioxidant capacity.

Wholegrain consumption elicits only minimal increases in systemic levels of phytochemicals in humans. Consumption of 100 g of boiled wheat bran increased postprandial plasma phenolic concentration by 5 μmol (60 min post ingestion) which represented <2% increase over baseline levels [98]. As these changes in circulating phenolic levels are minimal and of short duration it is unlikely that high intakes of wholegrains such as wheat can modulate systemic levels. Alternatively, alkylresorcinols, a class of phenolic lipids found at high levels in wheat and rye are relatively well absorbed within the small intestine (about 58%) [99], and as they are primarily transported in the serum in lipoproteins [100] they have a half life in serum of 5 h [101]. However, alkylresorcinols are rather weak antioxidants per se [102] and do not affect the susceptibility of LDL to oxidation ex vivo [103]. Wholegrains wheat, oats and barley are good dietary sources of betaine which can also contribute to improving antioxidant status as well as acting possibly as a methyl donor (transmethylation) and lipotrope [48,104,105]. The bran and aleurone layers of wheat are concentrated sources of betaine (~1% w/w) [104,106] and there is evidence in humans that the latter source is readily bioavailable [105].

It is important to consider how components of the diet may affect phytochemical bioavailability because cereal products are rarely consumed alone. Non-heme iron when consumed with cereals reduced the absorption of phenolics [107]. Milk may also reduce the absorption of phenolics [108], however other studies have also shown no impairment [109-111]. Flavonol absorption (in particular quercetin and its metabolites) may also be affected by a variety of dietary constituents such as ethanol, fat, and emulsifiers [96], but this observation is based on evidence from in vitro and animal studies and further research in humans is required.

b. Cereal bioprocessing for improving phytochemical bioavailability

Cereal bioprocessing is receiving increasing attention as a technique for purportedly improving the bioavailability of bound phytochemicals in grains. This technique utilises hydrolytic enzymes or enzyme cocktails to selectively release phytochemicals from the bran layer. However, there is very little evidence that cereal bioprocessing actually improves phytochemical bioavailability in humans. Recently Anson and colleagues [112] developed a bioprocessing technique whereby wheat bran undergoes a yeast fermentation and enzyme treatment procedure. When this wheat bran was incorporated into a wholemeal bread and consumed by volunteers the plasma concentrations of ferulic, vanillic and sinapic acids, and 3,4-dimethoxybenzoic acid were 2- to 3-fold higher than in the control bread [45]. Ferulic acid in particular increased in plasma to a maximal level of 2.5 μmol/L, which is considerably higher than baseline levels reported previously (5 to 30 nmol) [113]. The relevance of these changes in circulating phytochemical levels to metabolic health impact has yet to be demonstrated.

c. Phytochemical bioavailability in the large intestine: role of the microbiota

Microbial fermentation of cereal grains has the potential to increase the bioavailability of phytochemicals bound to the fibre matrix [114-117]. For instance, microbial esterases hydrolyse conjugated phenolic acids, such as those from wheat bran [118,119], potentially improving their absorption.[120]. In addition, ferulic acid from wheat bran has been shown to increase plasma antioxidant activity more effectively than pure ferulic acid in rats [121]. This highlights the important function cereals may have in delivering ferulic acid to the large bowel whereby enzymes from the microbiota cause the slow release of ferulic acid up to 24 h after its consumption. However, in humans there is limited evidence that large bowel fermentation contributes significantly to plasma phytochemical levels in the systemic circulation. A study by Kern et al. [113] showed that the absorption of wheat bran phenolics was limited essentially to the postprandial period. Plasma phenolics and metabolites of ferulic acid (hydroxycinnamic and diferulic acids) were at baseline levels 6 to 24 h after wheat bran consumption, suggesting that the microbial fermentation of the ingested wheat bran did not contribute to the systemic phenolic level. In addition, the authors also showed that diferulic acids (formed by microbial esterase digestion of ferulic acid) or their reduced dimers (formed by colonic microbiota hydrogenation reactions of diferulic acids) could not be detected in plasma or urine samples. In a study by Anson et al. [45] whole wheat bread increased plasma concentrations of two metabolites of ferulic acid (3-hydroxyphenylpropionic acid and phenylpropionic acid) 9 to 24 h after consumption by healthy volunteers. It is unlikely that these metabolites exert any biological affects systemically as the maximal plasma concentrations reached were only in the nanomolar range (100 nmol/L and 350 nmol/L respectively). The evidence suggests that the colonic microbiota contribute little to systemic levels of phenolic metabolites.

**Table 2:** Major wholegrain phytochemicals, factors affecting their bioavailability and suggested mechanisms for promoting health

**Phytochemical**	**Major grain sources**	**Food & dietary factors affecting bioavailability**	**Other factors that enhance bioavailability**	**Potential mechanisms of action**
Phenolics				
free	Oats	Milk	Unknown	Increase plasma total antioxidant capacity to directly mitigate oxidative stress
		Heme iron	Unknown	Indirect through cell signalling
bound	Wheat, barley, oats, rye	Grain structure	Bio-processing of grain	Increase plasma total antioxidant capacity to directly mitigate oxidative stress
			Colonic fermentation (limited evidence)	Indirect through cell signalling
Flavanoids	Wheat, barley	Grain structure	Unknown	Increase plasma uric acid levels which has reducing and free radical scavenging activities
				Improve glutathione radical scavenging system
Selenium	Wheat, barley, oats, rye	Not relevant as readily available	Not relevant as readily available	A cofactor for glutathione peroxidase, an enzyme that quenches reactive oxygen species

### Impact of wholegrain phytochemicals on metabolic health

Various blood and urine biomarkers are routinely used to determine the metabolic health benefits of wholegrain phytochemicals. For instance, plasma and urine levels of oxidised lipids provide an indirect measure of the capacity of cereal phytochemicals to protect circulating lipids from damage by reactive oxygen species. In addition, circulating levels of C-reactive protein and pro-inflammatory cytokines are indicative of low grade systemic inflammation, a hallmark of many metabolic diseases.

a. Oxidised lipids

There is some evidence supporting a role for wholegrain consumption in reducing oxidised lipids in plasma or urine. Kim et al. [122] showed that a mixture of brown and black rice when consumed for 6 wk by healthy adults reduced plasma thiobarbituric acid reactive substance (TBARS) levels. Jang et al. [19] also showed a reduction in oxidised plasma malondialdehyde (MDA) and urine 8-epi-prostaglandin F2α when subjects with coronary heart disease consumed a wholegrain powder mix (70 g/d) for 4 mo. Two other studies of shorter duration (2 and 6 wk) in which refined grain foods were replaced with wholegrain foods (7 to 8 servings/d) did not show any improvements in urinary levels of oxidised lipids.[123,124] LDL susceptibility to oxidation was also similar when healthy subjects consumed 250 g of rye or wheat bran bakery products for 6 weeks [103]. It is not clear from these later studies [103,123,124] whether the lack of an effect was due to the shorter duration of the interventions, differences in wholegrain type or differences in the type of biological fluid analysed (urine was analysed rather than plasma).

b. Antioxidant defence

The most promising evidence for wholegrain-rich diets improving blood-based antioxidant defence is through modulation of the glutathione radical scavenging system. This system utilises glutathione peroxidase to metabolise hydrogen peroxide to water by using reduced glutathione as a hydrogen donor [125]. The capacity for reduced glutathione to quench free radicals can be impaired if oxidised glutathione is not recycled back to glutathione by glutathione reductase, or if glutathione peroxidase activity is reduced [125]. An increase in reduced glutathione (21%) occurred 15 min after healthy subjects consumed an oat extract containing 1 g avenanthramide-enriched mixture and remained elevated (by up to 14%) for 10 h [126], a dose which far exceeds a level that could be achieved by consumption of wholegrain oats.. Alternatively, wholegrain dietary intervention studies showed that plasma glutathione peroxidase activity increased by 15% when subjects consumed brown and black rice for 6 wk [122] but decreased by 35% when subjects consumed a phytochemical-rich diet containing wholegrains for 4 wk [127]. These studies suggest that the type of wholegrain and duration of consumption is important in regulating glutathione enzyme status or redox state. A possible mechanism explaining the effect of wholegrains on glutathione balance comes from in vitro evidence that flavonoids alter the expression of genes responsible for the synthesis and regulation of glutathione (Table 2) [128,129]. There is further evidence from a dietary intervention study in humans that selenium improves glutathione peroxidase activity [130]. Subjects consuming brown or wholemeal bread made from wheat containing high levels of selenium increased whole blood glutathione peroxidase levels by 10% [131]. As most people in European countries have plasma selenium levels below the recommended level [132], wholegrain cereals with high selenium concentrations may offer an opportunity to improve glutathione status. Alternatively, Fardet [48] recently proposed that wholegrain wheat may increase glutathione levels through the supply of the sulfur amino acids methionine and cystine, which are precursors of glutathione. However, these amino acids are present in wholegrain wheat at low levels (0.5% of protein) [133], thus other dietary sources of sulphur amino acids such as egg and meat would presumably have a greater influence on circulating selenium levels.

There is limited evidence for whole grain-rich diets affecting copper-zinc superoxide dismutase (SOD), uric acid and tocopherol levels in the blood. Plasma SOD levels were unaffected in a study where subjects consumed black rice for 6 mo [134]. In contrast, another study showed that erythrocyte SOD levels were reduced when healthy adults consumed a phytochemical-rich diet containing wholegrains for 4 wk [127]. Plasma uric acid levels were increased (by 9%) in subjects who had consumed bread (200 g/d) made from inulin, linseed and soya fibre for 5 wk [135]. These findings are biologically significant in that uric acid accounts for up to 90% of plasma total antioxidant capacity [136]. Furthermore, high levels of flavanoids in some wholegrains are responsible for increasing plasma total antioxidant capacity as a result of stimulating uric acid levels rather than through the direct actions of flavonoids [136]. However, further research is required that investigates the impact of flavanoid-rich cereal consumption on uric acid levels and antioxidant status in healthy people as well as those with metabolic syndrome and T2D. Plasma α-tocopherol concentrations barely increase after consumption of wholegrains suggesting that this compound is of limited importance for the prevention of T2D [135], In addition, α-tocopherol contributes less than 2% of the antioxidant capacity of plasma [137], and a wholegrain-rich diet cannot provide the level of Vitamin E necessary to reduce oxidative stress in people with T2D (> 200 mg/d) [138] Furthermore, a review of human clinical trials concluded that vitamin E, and other common antioxidants, were not useful for managing diabetic complications [139].

c. Antioxidant capacity of blood

Most dietary intervention studies on wholegrains have used the ferric reducing antioxidant potential (FRAP) assay to determine plasma total antioxidant capacity. In two studies by the same group, meals consisting of approximately 100 g of wheat bran were fed to subjects and the postprandial change in plasma antioxidant status measured [98,140]. Both studies showed an increase but in the study by Beattie et al. [140] the magnitude of the response was only 4% (an increase of approximately 50 μmol of FRAP/L from a baseline of 1,204 ± 57.5 μmol of FRAP/L). It is not known whether this change is sufficient to protect against oxidative stress, a hallmark of metabolic syndrome and T2DM, and many other chronic diseases. In a study of longer duration (5 wk) fasted plasma FRAP levels did not change when subjects consumed bread (200 g/d) made with inulin, linseed and soya fibre, which had a 50% higher α-tocopherol content than the control German wheat-rye bread [135]. Although these studies show a somewhat promising result for wheat bran in improving plasma antioxidant capacity, the FRAP assay has some limitations. For instance, it does not account for the antioxidant capacity provided by blood proteins (as they too are extracted in sample preparation) and the assay is based on the reduction of iron which is considered too slow a measure of antioxidant potential [137,141]. Alternative antioxidant capacity assays, such as the one for plasma total antioxidant capacity (TAC), have been used to show an increase in plasma antioxidant capacity in subjects with coronary heart disease who consumed black rice for 6 mo [134]. Other total antioxidant capacity assays, such as the Oxygen Radical Absorbance Capacity (ORAC) and Trolox Equivalent Antioxidant Capacity (TEAC), may be useful for evaluating radical scavenging, however they are not suitable for assessing lipid peroxidation inhibition [142]. Thus future studies should deploy a combination of different antioxidant capacity assays and the results interpreted in the context of changes in plasma lipid and protein oxidative stress biomarkers and clinical outcomes.

d. Anti-inflammatory actions

There is growing evidence supporting a reduction in pro-inflammatory markers in people consuming higher levels of wholegrains and/or cereal fibre. For instance, cereal fibre intakes (> 8.8 g/d), but not total fibre, were associated with significantly lower plasma cytokine levels in healthy adults [143]. Intervention trials provide evidence that plasma cytokines or C-reactive protein were reduced after consumption of bakery products containing rye bran [144], bread made from whole wheat with bioprocessed bran [45] or a black rice pigment fraction [134].

The fibre component of wholegrains is often associated with having favourable effects on pro-inflammatory markers including C-reactive protein and interleukin-6 [145,146]. In particular, the fermentation of cereal fibre in the large bowel produces short chain fatty acids (SCFA) that bind to G-protein coupled receptors, inhibiting transcription factor Nfκβ and thereby increasing the threshold for an inflammatory response in the colonic mucosa [147]. The anti-inflammatory actions of SCFA may extend beyond the large bowel as these bacterial metabolites are readily absorbed by colonocytes [148]. However, SCFA concentrations in the systemic circulation are low (<0.2 mM) as most SCFA absorbed from the lumen of the gut are metabolised extensively by the gut mucosa and the liver. Furthermore, consumption of fermentable dietary fibres produces only a modest rise in plasma SCFA levels [149]. Whether these modest levels of circulating SCFA are sufficient to prevent or attenuate the elevated inflammatory status of individuals with diabetes and related disorders is yet to be established and deserves further investigation. Dietary fibre may help prevent chronic inflammation by also reducing circulating levels of lipoplysaccharides (LPS), which are known to contribute to the development of obesity-related inflammatory liver diseases [150-152]. The consumption of prebiotics has been shown to restrict the translocation of LPS from the large bowel of mice fed a high fat diet, resulting in reduced markers of inflammation in adipose tissue [153]. However, the relevance of these findings for humans is not yet clear.

## Conclusions

Evidence from postprandial and medium-term intake studies suggest that the phytochemical component of cereals provides limited benefit for preventing oxidative stress and development of T2D. Wholegrain consumption may increase postprandial plasma phenolic levels but the response is modest and transient. Whether this effect is sufficient to bolster antioxidant defences and improve health outcomes has not been established. Although there is growing interest in the colonic microbiota and bio-processing for increasing phytochemical bioavailability the improvements so far are small and have not improved markers of clinical relevance for reducing risk of T2D. Future dietary intervention studies seeking to establish a direct role of phytochemicals in mediating the metabolic health benefits of wholegrains, and their potential for mitigating disease progression, should consider using varieties that deliver the highest possible levels of bioavailable phytochemicals in the context of whole foods and diets. Both postprandial and prolonged responses in systemic phytochemical concentrations and markers of inflammation and oxidative stress should be monitored and along with changes related to health outcomes in healthy individuals as well as those with metabolic disease.

## Abbreviations

FAE: Ferulic acid equivalent; FRAP: Ferric reducing antioxidant potential; LPS: Lipopolysaccharide; MDA: Malondialdehyde; ORAC: Oxygen Radical Absorbance Capacity; SCFA: Short chain fatty acid; SOD: Superoxide dismutase; TAC: Total antioxidant capacity; TBARS: Thiobarbituric acid reactive substance; TEAC: Trolox Equivalent Antioxidant Capacity; T2D: Type-2 diabetes Mellitus.

## Competing interests

There are no conflicts of interest.

## Authors’ contributions

DPB and ARB participated in the acquisition, analysis and interpretation of data, and drafting of the manuscript. Both authors read and approved the final manuscript.
